# ‘I have to remind myself that everyone’s search is different’: experiences and outcomes of searching and not searching for donor connections among donor conceived adults

**DOI:** 10.1093/humrep/deae210

**Published:** 2024-09-17

**Authors:** S Zadeh, C Jones, V Jadva

**Affiliations:** School of Psychology, University of Sussex, Brighton, UK; Social, Genetic and Developmental Psychology Centre, King’s College London, London, UK; Department of Psychology, City, University of London, London, UK

**Keywords:** donor conception, donor connections, donor siblings, donor, sperm donation

## Abstract

**STUDY QUESTION:**

What are the experiences and outcomes of donor conceived adults who are actively searching for, open to contact with, or not searching for donor connections?

**SUMMARY ANSWER:**

Most participants were actively searching or open to contact, and 67% had found or been found by a connection; finding or not finding experiences were complex.

**WHAT IS KNOWN ALREADY:**

There is variation among donor conceived individuals in their interest in donor connections. Individual reasons for searching for connections, and which donor connections are searched for, also vary. Most research studies have focussed on individuals who are actively searching for their donor or donor siblings. Global increases in direct-to-consumer DNA testing and social media participation mean that connections may be made to individuals unaware of their (or their relatives’) involvement with donor conception. These social and technological changes have also increased the chances of donor conceived individuals being contacted without expecting or desiring contact.

**STUDY DESIGN, SIZE, DURATION:**

This study included 88 donor conceived adults, in the UK, who participated in an online multi-method survey between January and August 2022. The survey was designed in consultation with staff and volunteers from the UK’s largest community networks for donor conception families (Donor Conception Network, DCN) and donor conceived people (Donor Conceived Register Registrants’ Panel, DCRRP). It was piloted by five donor conceived people before its launch. Participants were recruited with assistance from DCN and DCRRP, via social media, university mailing lists, and snowballing.

**PARTICIPANTS/MATERIALS, SETTING, METHODS:**

Participants were mostly female (n = 65, 74%) and sperm donor conceived (n = 79, 90%). Of the 88 participants, 39 (44%) were actively searching for their donor connections, 44 (50%) were open to contact but not actively searching, and 5 (6%) were not searching. Questions were closed (yes/no, rating scale, or multiple choice) or open-ended, addressing experiences of donor conception, searching for connections, and finding or not finding connections. Data were analysed both quantitatively and qualitatively.

**MAIN RESULTS AND THE ROLE OF CHANCE:**

Quantitative results showed no differences between the groups on any demographic variables or in when or how they found out about being donor conceived, and no differences between active searchers and those open to contact in whether they had found their donor connections. Significant differences were found between groups in their interest in their genetic history and the perceived importance of genetics to their sense of identity, with active searchers being more interested and rating this as more important than those open to contact. Methods of searching significantly differed across groups, with active searchers using genetic testing and social media more than those open to contact. 59 participants across all groups (active searchers (n = 29, 74%), open to contact (n = 27, 61%), not open to contact (n = 3, 60%)) had found or been found by a donor connection. Experiences of finding or not finding donor connections among participants actively searching or open to contact were captured by the theme complexities, with six subthemes: uncertainties in searching and relating; searching as open-ended; different donor connections, different experiences; expectations and realities; searching and finding or not finding as catalysing change; and experiences of other donor conceived people.

**LIMITATIONS, REASONS FOR CAUTION:**

Most participants were members of relevant community organizations. As is common in research in this area, the sample was mostly female and conceived using donor sperm. Donor conceived people who are disinterested in donor connections may be unlikely to participate in research on this topic.

**WIDER IMPLICATIONS OF THE FINDINGS:**

The nature and impact of the search process itself should be considered when developing appropriate mechanisms of support for all donor conceived people, regardless of whether they are actively searching for connections or not. Further research should seek to better understand how donor conceived people with varying levels of interest in searching for donor connections differ from one another.

**STUDY FUNDING/COMPETING INTEREST(S):**

This study was funded by the UK Economic and Social Research Council [New Investigator Award ES/S015426/1]. The authors have no competing interests to declare.

**TRIAL REGISTRATION NUMBER:**

N/A.

## Introduction

Increasing numbers of donor conceived individuals are searching for their donor connections, either their donor or their donor siblings (others conceived through the same donor as themselves and who have different parent/s). In 2005, the UK changed its laws regulating gamete donation from anonymous donation to identifiable donation, such that as of October 2023, donor conceived people conceived on or after 1 April 2005 can access information about their donor’s identity at age 18 years. At this age, they can also request information about the identity of any donor siblings who have also expressed an interest in contacting their donor siblings. Given the move towards openness of donor identity and donor connections in some jurisdictions, and the greater ease of searching for donor connections through other means such as direct-to consumer DNA testing, understanding the various experiences of donor conceived people with regards to searching for and finding their donor and donor siblings is both timely and important.

Researchers have reported variation in donor conceived individuals’ interest in their donor connections. Some studies have found a strong desire to know donor connections amongst donor conceived people, and that being unable to find these connections can lead to feelings of frustration over a lack of information (Cushing, 2010; Turner and Coyle, 2020). In contrast, a longitudinal study of young adults born following gamete donation and surrogacy in the UK found that among those not in contact with their surrogate or donor, most were not actively searching for them (Jadva *et al.*, 2023). A recent systematic review by Indekeu *et al.* (2021) highlighted potential associations between interest in and searching for donor connections and gender, age of disclosure, family type, and family relationships. Jadva *et al.* (2010) found that motivations for searching for donor siblings were associated with age of disclosure, with those who found out about their conception after the age of 18 years more likely to be searching for medical reasons and to gain a better understanding of themselves than those told prior to age 18 years, who were more likely to cite curiosity as their main reason for searching. Other studies have also found that donor conceived individuals with identifiable donors and who learned of their conception later in life were significantly more interested in information about their heritage and medical background, and in establishing contact with the donor’s family, than were those who had experienced earlier disclosure (Lampic *et al.*, 2022). Thus, the degree to which donor conceived individuals wish to identify either their donor or their donor siblings, and their reasons for doing so, vary. Differences have also been found in the meanings individuals make of donor connections, once established, to the donor or donor siblings (Frith *et al.*, 2018; Newton *et al.*, 2023), although little is yet known about the factors that might underpin this variance.

Donor conceived individuals who are interested in or seek contact with the donor have been shown to want not only to learn more about them (e.g. the donor’s appearance, interests, reasons for donation, and medical information), but also to satisfy feelings of curiosity, and to answer questions about their own identity (see Indekeu *et al.*, 2021, for a systematic review). The two most reported motivations for interest in or contact seeking with donor siblings are to learn donor-related information (e.g. donor siblings’ appearance, interests, and character) and to form new relationships (Indekeu *et al*., 2021). Interest may be general (i.e. in any donor connections), or it may be specific (i.e. in either the donor or donor siblings, but not both, see Jadva *et al.*, 2010; Persaud *et al.*, 2017). Searching may also result in connections that were not actively sought (Cushing, 2010). Unlike previously, where donor connections often resulted from actively searching through donor-linking websites (Jadva *et al.*, 2010) or publicly funded registers and services such as the Donor Conceived Register (DCR) in the UK (Crawshaw *et al.*, 2016), the recent rise of direct-to-consumer DNA testing alongside increased use of social media has also opened up the possibility of making contact with donor connections who are unaware of their (or their relatives’) involvement with donor conception (Crawshaw, 2018; Guerrini *et al.*, 2022; Bauer and Meier-Credner, 2023; Gilman *et al.*, 2024). Direct-to-consumer DNA testing has also increased the chances among donor conceived individuals of being contacted without desiring contact.

Qualitative research studies have shown that finding donor connections may lead to greater self-understanding and a sense of belonging (Klotz, 2016; Persaud *et al.*, 2017; Frith *et al.*, 2018; Daniels, 2020; Scheib *et al.*, 2020). Although mostly positive experiences of contact with the donor and/or donor siblings have been found, less positive contact experiences and relationships have also been documented (Jadva *et al.*, 2010; Scheib *et al.*, 2020, 2023). Research on donor sibling connections has shown that these connections may be difficult to navigate because of a mismatch in the expectations of the different parties involved, a mismatch in donor siblings’ level of interest in the donor, and/or physical and/or emotional distance (Jadva *et al.*, 2010; Blyth, 2012; Scheib *et al.*, 2020; Hertz, 2022; Indekeu *et al.*, 2022). Discovering the existence of large same-donor networks and meeting multiple donor siblings have also been shown to be challenging experiences (Frith *et al.*, 2018; Hertz, 2022; Indekeu *et al.*, 2022; Bolt *et al.*, 2023).

Given the variation in donor conceived individuals interests in and reasons for searching, and the varying importance among donor conceived individuals of knowledge of and contact with the donor and/or donor siblings, the present study aimed to explore experiences and outcomes of searching (and not searching) among donor conceived adults who were actively looking for their donor connections, those open to contact but not actively searching, and those not desiring contact (but who may be found). This is to our knowledge the first study to have examined the search experiences of these groups together.

## Materials and methods

### Method

Data for this study are from a larger survey-based investigation of donor conceived adults in the UK. The survey was designed in consultation with staff and volunteers from the UK’s largest community networks for donor conception families (Donor Conception Network, DCN) and donor conceived people (Donor Conceived Register Registrants’ Panel, DCRRP, now Donor Conceived UK). It was piloted by five donor conceived people prior to launch, and was live, via Qualtrics, between January and August 2022.

The survey was advertised by the DCN and DCRRP via mailing lists and social media. It was also circulated by the research team and others on social media and university mailing lists. Snowball sampling was also employed. The inclusion criteria for the study were: born through gamete donation (oocyte, sperm, or embryo donation); aged over 18 years; and living in the UK. Ethical approval was awarded by the UCL IOE Research Ethics Committee. The study was also approved by the Donor Conception Network Research Ethics Committee. All participants provided written consent to take part in the survey.

### Participants

A total of 88 donor conceived adults completed the survey. Of these, 39 participants (44%) described themselves as actively searching for donor connections, 44 participants (50%) described themselves as open to contact but not actively searching for donor connections, and 5 participants (6%) described themselves as not searching for donor connections. The majority of participants found about the study through the DCRRP (n = 45, 51%) or DCN (n = 22, 25%).

Participants were aged between 18 and 70 years (Mean = 34.27 years, S.D. = 10.95 years) and living in the UK. Given the aims of the overall study, which looked at identity and wellbeing among donor conceived people, particularly those in young adulthood, a wealth of demographic data was collected. Most participants were female (n = 65, 74%) and had been conceived using donor sperm (n = 79, 90%), as is common in research on this topic (Indekeu *et al.*, 2021). The sample was majority white (n = 87, 99%), heterosexual (n = 65, 74%), non-religious (n = 67, 76%), and, in terms of education, had a first degree or higher (n = 60, 68%). The majority of participants (n = 74, 84%) described their parents’ situation at the time of their conception as part of a heterosexual couple, and most participants (n = 53, 60%) had siblings (including step-siblings and half-siblings, excluding donor siblings). Demographic information by search status is reported in Table 1. There were no differences between active searchers and those open to contact on any of the demographic variables.

**Table 1.** Sample characteristics by search status.

	Actively searching		Open to contact		Not searching	
	N = 39		N = 44		N = 5	
	X	SD	X	SD	X	SD
**Age**	33.97	10.20	34.64	11.74	33.40	11.74
	**N**	**%**	**N**	**%**	**N**	**%**
**Gender** ^a^						
Female	30	77.3	31	70.5	4	80.0
Male	7	20.5	11	25.0	0	0
Nonbinary	1	2.6	2	4.6	1	20.0
Transgender	1	2.6	0	0	0	0
**Sexual orientation**						
Straight or heterosexual	27	69.2	34	77.3	4	80.0
Gay or lesbian	3	7.7	4	9.1	1	20.0
Bisexual	6	15.4	3	6.8	0	0
Other	2	5.1	3	6.8	0	0
Missing	1	2.6	0	0	0	0
**Relationship status**						
Married/civil partnership	13	33.3	21	47.7	2	40.0
In a relationship	13	33.3	14	31.8	2	40.0
Single	13	33.3	9	20.5	1	20.0
**Ethnicity**						
White English/Welsh/Scottish/Northern Irish/British	37	94.9	41	93.2	5	100
White other	2	5.1	2	4.6	0	0
Mixed/multiple ethnic groups	0	0	1	2.3	0	0
**Religion**						
No religion	32	82.1	32	72.7	3	60.0
Christian	6	15.4	11	25.0	1	20.0
Jewish	0	0	1	2.3	1	20.0
Buddhist	1	2.6	0	0	0	0
**Family type at conception**						
Heterosexual couple	35	89.8	35	79.6	4	80.0
Single mother	1	2.6	4	9.1	0	0
Same-sex female couple	3	7.7	3	6.8	1	20.0
Other	0	0	2	4.5	0	0
**Education**						
GCSEs	2	5.1	4	9.1	0	0
A-levels	4	10.3	7	15.9	1	20.0
Undergraduate degree	14	35.9	16	36.4	0	0
Postgraduate degree	14	35.9	13	29.5	3	60.0
Diploma	4	10.3	3	6.8	1	20.0
Other	1	2.6	1	2.3	0	0
**Employment status**						
Employed	28	71.8	31	70.5	2	40.0
Unemployed	2	5.1	1	2.3	0	0
Studying	2	5.1	4	9.1	1	20.0
Employed and studying	2	5.1	3	6.8	1	20.0
Other	5	12.8	5	11.4	1	20.0
**Method of conception**						
Sperm donation	38	97.4	37	84.1	4	80.0
Oocyte donation	1	2.6	5	11.4	1	20.0
Embryo donation	0	0	2	4.5	0	0
**Siblings (excluding donor siblings)**						
Siblings	23	59.0	26	59.1	4	80.0
No siblings	16	41.0	18	40.9	1	20.0

a Participants could select multiple responses.

### Measures

The present paper reports from a section of the survey designed to capture donor conceived people’s experiences and outcomes of searching for donor connections. Questions were both closed (yes/no, rating scale, or multiple choice, with an open-text option for a response not listed) and open-ended. Questions could be skipped.

#### Experiences of and engagement with donor conception

Information was obtained on: (i) whether participants could remember the age they learnt about their donor conception (yes, always known or too young to remember, not sure); (ii) if yes, the age participants learnt about their donor conception; (iii) how they learnt about their donor conception, selecting from one or more possible options (told by a parent, told by a sibling, told by a family member other than a parent or sibling, from a genetic test, learnt another way); (iv) whether participants had told other people about being donor conceived (yes, no); (v) how often participants had conversations about being donor conceived (never, less than once a year, a few times a year, once a month, several times a month); and (vi) whether participants were part of a support group/organization for donor conceived people (yes, no).

#### Interest in and perceived importance of genetic information

Information was obtained on: (i) how interested participants were in their genetic history (not at all interested, not interested, neutral, interested, very interested); and (ii) how important participants felt that genetics was to their identity (not at all important, not important, neutral, important, very important).

#### Searching for donor connections

An open-ended question asked participants: (i) their reasons for searching or not searching at present. Participants who were actively searching or open to contact were asked: (ii) how they had searched or made themselves open to contact, by selecting from one or more options (via the HFEA, a network for donor conceived people, genetic testing, genetic genealogists, social media, not listed (please describe)); and (iii) how frequently they searched (look frequently, look occasionally, not checking but have registered on a DNA testing website, not checking but have registered on other platforms, e.g. DCR).

#### Experiences of finding or not finding donor connections

All participants were asked: (i) if they had been found by a donor connection (yes, no); and (ii) if yes, who, selecting from one or more options (sperm donor, oocyte donor, donor siblings/half-siblings who share the same donor as you, donor’s children, other genetic relatives, e.g. donor’s parents, donor’s siblings, not listed (please specify)). Participants who were actively searching or open to contact were asked: (iii) who they had found, by selecting from one or more options (sperm donor, oocyte donor, donor siblings/half-siblings who share the same donor as you, donor’s family, not listed (please specify)); (iv) for each connection, whether they had made contact (yes, no); (v) whether their expectations of searching or being open to contact had been met (definitely not, not really, in some ways, mostly, definitely); and (vi) an open-ended question asking for further details about their answer to (v). An open-ended question to participants who were actively searching or open to contact but who had not made a connection addressed: (vii) their feelings about not having yet made any connections. Open-ended questions to all participants addressed: (viii) how they felt about the donor, compared to how they felt before searching for or before any contact with the donor; and (ix) how they felt about donor siblings, compared to how they felt before searching for or before any contact with donor siblings.

### Analysis

#### Quantitative analysis

Data examining experiences of and engagement with donor conception, interest in and perceived importance of genetic information, and searching for donor connections were analysed using chi-square analysis for categorical data and independent samples Mann–Whitney *U*-tests to compare differences between active searchers and those open to contact but not actively searching. As the group of participants not searching was small (n = 5), they were not included in the statistical analysis, although the descriptive data are presented in the tables for comparison. To understand experiences of finding or not finding donor connections, the groups of active searchers and those open to contact were further divided by whether or not they had found their donor connections. Kruskal–Wallis tests were conducted for these analyses and *post hoc* comparisons were carried out using Mann–Whitney *U*-tests with a Bonferroni-adjusted alpha level of 0.008.

#### Qualitative analysis

Owing to the varied nature of the qualitative data collected, two types of qualitative analysis were performed, addressing (i) reasons for presently searching or not searching for connections and (ii) experiences of finding or not finding connections, respectively.

Qualitative content analysis (Schreier, 2012), which involved creating a coding frame of categories from all relevant responses to the question about reasons for presently searching or not searching, was used to understand participants’ reasons across groups. The question was answered by 85 participants, of whom 10 participants gave responses that did not address the question (i.e. describing the nature of their search, or giving reasons for searching in the past) and were excluded. The remaining 75 responses were coded, with responses coded more than once where appropriate (i.e. where multiple reasons given). Categories (corresponding to reasons) were refined throughout the analytic process, resulting in the identification of a total of 22 categories, each relating to a discrete reason for searching or not searching at present.

To understand in greater depth participants’ thoughts, feelings and experiences of finding or not finding donor connections, all open-ended questions relating to this topic were analysed qualitatively according to the principles of thematic analysis (Braun and Clarke, 2001). There were 47 responses to the question about feelings about the donor compared to before searching or before contact, 50 responses to the question about feelings about siblings compared to before searching or before contact, 68 responses to the question about expectations, and 27 responses to the question about having not made any connections (i.e. excluding all missing data or ‘not applicable’ responses). Responses were coded inductively (that is, from the data rather than using a predefined coding frame) by participant (rather than by question), with both descriptive (e.g. ‘finding not believed to be likely’) and analytic (e.g. ‘ambivalence’) codes generated, resulting in a total of 21 initial codes. The codes and coded extracts were then read and re-read, and a total of one theme and six subthemes were identified.

## Results

### Experiences of donor conception

Table 2 shows the experiences of donor conception for each group. There were 66 participants who provided an age at which they had found out about their conception which ranged from 6 to 50 years of age (Mean = 25.41, SD = 11.30). There was no difference in the age at which active searchers and those open to contact had found out about their donor conception. There was no difference between groups in the proportion of donor conceived people who had found out about their donor conception from their parents, a sibling, another family member, a genetic test, or through other means. All participants had told others that they were donor conceived, and there was no difference between active searchers and those open to contact in how frequently they discussed their donor conception with others. There was also no group difference in whether participants were members of a support group or organization for donor conceived people.

**Table 2.** Experiences of donor conception by search status.

	Actively searching		Open to contact		Not searching	
	Mean	SD	Mean	SD	Mean	SD
**Age found out about conception**	24.48	10.14	26.34	12.41	25.00	14.00
	**N**	%	**N**	%	**N**	%
**Method of finding out about conception** ^a^						
Told by a parent	27	69.2	35	79.5	3	60
Told by a sibling	0	0	0	0	5	100
Told by another family member	0	0	1	2.3	0	0
From a genetic test	6	15.4	5	11.4	1	20
Other (e.g. accidental discovery or unplanned disclosure)	7	17.9	5	11.4	1	20
**Frequency of conversations about donor conception**						
Very often	19	48.7	16	36.4	3	60
Sometimes	8	20.5	9	20.5	1	20
Occasionally	10	25.6	17	38.6	1	20
Rarely	2	5.1	2	4.5	0	0
**Donor conception support group/organization member**						
Yes	33	84.6	35	79.5	3	60
No	6	15.4	9	20.5	2	40

a Participants could select multiple responses.

### Interest in and importance of genetic information

There was a significant difference between groups in how interested they were in learning about their genetic history (*U* = 1141.50, *P* = 0.001), with active searchers rating this higher (Median = 5, IQR = 0) than those open to contact (Median = 5, IQR = 1). There was also a significant difference between groups in how important genetics was to their sense of identity (*U* = 1148.50, *P* = 0.004). with active searchers rating this as more important (Median = 5, IQR = 1) than those open to contact (Median = 4, IQR = 2).

### Searching for donor connections

As can be seen in Table 3, most participants who were either actively searching or open to contact had used genetic testing. Half of the sample had used the DCR and 41% had searched or were open to contact through the UK regulator, the HFEA. There was a significant difference between active searchers and those open to contact in the proportion of people who had searched using genetic testing (*X*^2^ (1,83) = 8.325, *P* = 0.004) with active searchers more likely to have used this method. There was also a significant difference between groups in the use of social media (*X*^2^ (1,83) = 4.100, *P* = 0.004) with active searchers stating that they had used social media more than those open to contact. There was a non-significant trend suggesting greater use of different methods by active searchers compared to those open to contact (*U* = 666.50, *P* = 0.070). As would be expected, there was also a significant difference between groups in how frequently they were looking for donor connections (*X*^2^ (1,83) = 19.126, *P* = 0.004), with active searchers looking more frequently than those open to contact.

**Table 3.** Searching for donor connections by search status.

	Actively searching		Open to contact	
	N	%	N	%
**Method of searching/openness to contact** ^a^				
Human Fertilisation and Embryology Authority	15	38.5	19	43.2
Donor Conception Network	12	30.8	12	27.3
Donor Conceived Register	19	48.7	23	52.3
Genetic testing	37	94.9	31	70.5
Genetic genealogists	2	5.1	3	6.8
Social media	17	43.6	10	22.7
Other	1	2.6	0	0
**Frequency of searching**	N	%	N	%
Look frequently	14	35.9	4	9.0
Look occasionally	18	46.2	13	29.6
Not checking but have registered on a DNA testing website	4	10.2	12	27.3
Not checking but have registered on other platforms	2	5.1	14	31.8
Missing data	1	2.6	1	2.3

a Participants could select multiple responses.

A total of 22 reasons for presently searching or not searching for donor connections were given (see Table 4). Participants in all three groups mentioned identity and belonging (n = 19), family relationships (n = 11), and having found connections (n = 4) as reasons for presently searching or not searching. Examples of responses from participants who mentioned identity and belonging included:

*I am keen to find out my identity, who am I really?* (Active searcher, not connected)

*I am curious about all sorts, not least of all myself.* (Open to contact, connected)

*I am comfortable with my identity, and I belong to such a supportive family that I do not feel the need to expand my family in that way.* (Not searching, not connected)

**Table 4.** Reasons for searching/not searching at present, by search status.

Reason for searching/not searching	Actively searching	Open to connections	Not searching
Identity and belonging	12	6	1
Genetic information and medical history	6	1	0
Want to find more siblings	6	0	0
Family relationships	5	3	3
Curiosity/general desire to know	5	4	0
Want as much information as possible	5	0	0
Looking for either donor or siblings (have identified the other)	3	3	0
Time is running out	3	0	0
Open to contact from others	2	3	0
Found (so no longer searching as before)	1	1	2
Worried about the outcome	0	7	1
Too busy	0	6	0
Not ready/not sure	0	3	0
Donor doesn’t want contact	0	2	1
Dissuaded by false or no matches	0	3	0
Unlikely to find anyone	0	2	0
Too expensive	0	2	0
Tiring	0	2	0
Other (each of which n = 1)	1	3	0

Examples of responses from participants who mentioned family relationships included:

*I’m looking for people like me—this is because I spent my early life feeling alone and different within my family.* (Active searcher, connected)

*I am open to making connections because I did not grow up with siblings, and do not have any close cousins. I am curious about similar traits a donor sibling may have. Finding a donor sibling would be interesting, but I would not be really upset if I could not find any.* (Open to contact, not connected)

*I am not interested in having contact with my donor or donor siblings. I have a family that I love and hardly have enough time to see them as it is. I don’t feel the need to connect with the donor side of my family…I’m open to feeling differently in the future but at this time in my life it is not something that interests me.* (Not searching, connected)

Examples of responses from participants who had found a connection and were therefore no longer searching as before included:

*I have searched and hence discovered my biological father’s identity and now am part of a family of DC half-siblings. Therefore I no longer need to search so actively as before.* (Active searcher, connected)

*I have found my biological father but have not yet made contact, therefore no longer searching. I will write a letter soon. I have written a number of drafts but these things take time. Especially given I was conceived anonymously in 1980.* (Open to contact, connected)

*I do not need to search as within a couple of hours of learning I was DC* [via DNA testing] *I was put into a WhatsApp group with 8 of my half siblings and donor!* (Not searching, connected)

### Experiences of finding or not finding donor connections

There were 59 participants across all groups (active searchers (n = 29), open to contact (n = 27), not open to contact (n = 3)) who had found or been found by a donor connection. There was no significant difference between active searchers and those open to contact in whether they had found their donor connections. Almost all participants who had found donor connections (100% of those actively searching, and over 92% of those open to contact) had used genetic testing. Participants who were actively searching or open to contact had connected with their donor and donor siblings, with smaller proportions finding other connections. All but five participants had contacted their connections once found; one reported that they had not made contact and four (two actively searching, and two open to contact) reported that they were considering making contact in the future. Participants who were not searching, but had been found, had been identified by donor siblings (see Table 5).

**Table 5.** Donor connections by search status.

	Actively searching and found		Open to contact and found		Not searching	
	N = 29		N = 27		N = 5	
	N	%	N	%	N	%
**Who have you found?**						
Donor	21	72.4	14	51.9	–	–
Donor siblings	20	69.0	17	63.0	–	–
Donor’s family	20	69.0	11	40.7	–	–
Other (e.g. cousins, half-nephew)	1	3.4	2	7.4	–	–
**Have you been contacted by someone who identified you as a donor connection?**						
Yes	14	48.3	11	40.7	3	60
No	15	51.7	16	59.3	2	40
**Who identified you?**						
Sperm donor	1	3.4	1	3.7	0	0
Oocyte donor	0	0	0	0	0	0
Donor siblings^a^	11	37.9	10	37.0	3	60
Donor’s children	0	0	0	0	0	0
Other genetic relatives, e.g. donor’s parents, donor’s siblings	3	10.3	1	3.7	0	0
Response did not address question	0	0	1	3.7	0	0

a One participant in the not searching group, who was identified by a donor sibling, was also in contact with their donor.

There was a significant difference between groups in whether their expectations of searching or being found had been met (*H* (3, *n *=* *83) = 15.982, *P *=* *0.001). Active searchers who had found their donor connections were more likely to feel that their expectations had been met (Median = 3, IQR = 2) than those who were actively searching but had not found connection (Median = 3, IQR = 1). (*U* = 55.00, *P* = 0.003) and those who were open to contact but had not found their donor connections (Median = 4, IQR = 2) (*U* = 110.50, *P* = 0.001).

Participants’ open-text responses relating to their experiences of finding or not finding were characterized by one theme, complexities, and six subthemes: uncertainties in searching and relating; searching as open-ended; different donor connections, different experiences; expectations and realities; searching (and finding or not finding) as catalysing change; and experiences of other donor conceived people (including donor siblings). The theme and subthemes were present in the responses of participants who were actively searching or open to contact and who had both found or not found connections, i.e. irrespective of search status and search outcome. The subtheme of experiences of other donor conceived people, including donor siblings, was also present in the responses of participants in the not searching group. Similarities and differences between groups are highlighted below, along with overlaps between different subthemes, where relevant.

#### Uncertainties in searching and relating

Several participants highlighted the uncertainties involved in making or not making connections. As one participant explained: ‘*I have narrowed the donor down to one of three brothers but it still feels very strange as nothing is confirmed*’ (Active searcher, not connected). Another participant stated: ‘*I question whether or not my donor understands that anonymity can be removed if requested by themselves, the donor*’ (Active searcher, not connected). This participant, like several in the study, also expressed feeling uncertain about their donor siblings’ knowledge of their conception.

Another active searcher, who expressed similar concerns about donor siblings, also expressed confusion about the lack of response from their donor, whom they had contacted:

*I wonder why he hasn’t replied. I wonder why he cared enough about my parents having a baby to bother donating, but then doesn’t care enough about the baby once it grows up to reply to them. I am especially confused as to why he would put his DNA on a site if he wasn’t going to reply. It feels cruel.* (Active searcher, connected)

Some participants explained that the uncertainties involved in searching had prohibited them from searching or initiating contact, or had otherwise featured in their thoughts:

*I haven’t started officially looking yet. I know that I cannot control the outcome, and the fear of worst case scenarios seems to outweigh the possibility of finding information.* (Open to contact, not connected)

*I’m quite happy in control of the situation at the moment—keeping them all as a fantasy. The reality may be very different and I may be rejected which is a lot to deal with. I’m not ready for that quite yet.* (Open to contact, connected)

For some participants who had found connections, it was an ambivalent experience, even in cases where matching was described as broadly positive:

*It’s difficult. On the one hand, it is wonderful to know them and I’m so happy to have found them. They are really lovely people too. However, it can be awkward. We are strangers after all and it’s such a strange situation that we haven’t really been prepared for. In the initial stages there’s definitely a fear of rejection and even later it’s tricky trying to navigate these relationships.* (Active searcher, connected)

*My donor has four children he brought up. I’ve yet to meet or make connection with them. They are happy to know of my existence and I’ve been told they are open to connecting sometime. I’m just not sure how to start that relationship*. (Open to contact, connected)

#### Searching as open-ended

Related to the previous subtheme, several participants stressed the open-ended nature of searching, irrespective of whether they had found connections. For some, this was an ongoing uncertainty:

*I think I will be forever searching for siblings as bio father donated close to 200 times in a year. Glad I have managed to locate some of them so far.* (Active searcher, connected)

*I would like to find someone. As I am signed up to Ancestry, I could potentially have an email notification for a match at any time, however over the last year there has been no close or identifiable matches.* (Open to contact, not connected)

As the above quotations suggest, the fact that matches could emerge at any time was perceived in different ways by different participants. One participant explained that ‘*I haven’t given up hope yet…but it’s horrible whilst I’m waiting*’ (Active searcher, connected), while another described that it created ‘*an odd feeling*’ (Active searcher, connected).

#### Different donor connections, different experiences

Several participants explained that they had different experiences with different donor connections. Some participants described a positive experience with the donor, but less positive experiences with their sibling/s. One active searcher explained that ‘*My bio father and I have become close and see each other frequently*’, describing their experience with him as ‘*very positive*’. Regarding siblings, however, they explained that they felt ‘*a little deflated. The contact has been minimal and* [I] *thought we may be closer*’. Other participants described a negative experience with their donor, but positive experiences with their sibling/s. One participant who was open to contact stated that they felt ‘*angry, hurt*, [and] *rejected*’ by the donor, but ‘*happy* [and] *bonded*’ with siblings. A few participants explained that the donor had died before they had an opportunity to make contact, but that they had established positive relationships with donor siblings and, in some cases, members of the donor’s family.

Several participants also described different experiences with different donor siblings:

*Some of the siblings I connected with on Ancestry are aware of their DC status but do not wish to know anything or have a relationship. I can appreciate that and respect that boundary. For the three girls I do have a relationship with, we are building and establishing how things work for us all and it’s an evolving journey.* (Active searcher, connected)

*Donor siblings have been either very happy with the family they grew up with and not very interested or have been very unhappy with their families and almost “cling” to the idea of you as a half sibling.* (Open to contact, connected)

#### Expectations and realities

Among participants who had identified connections, feelings of low and high expectations were expressed, irrespective of current search status (i.e. active/open).

*I didn’t have high expectations—I recognised that reactions to being donor conceived are deeply personal, so I was prepared for rejection or denial. I’ve had a good experience—I’m fortunate in that sense.* (Active searcher, connected)

*My expectations were that I hoped I would find the donor and/or anyone else in his family and I did. I hoped it would be a positive experience and it was.* (Active searcher, connected)

*I found it to be a really emotional process, obviously. My expectations were low, I had read enough to know I was unlikely to find my donor and that if I did it was likely he wouldn’t want contact. In that sense I have been incredibly lucky. I have met my donor several times, call him Dad and have a wonderful connection and relationship with him. In that sense, it was far above and beyond any expectations I had.* (Open to contact, connected)

Several participants who had not made connections explained that they were disappointed not to have done so. This was the case both for those who described themselves as actively searching and those who said they were open to connections, suggesting a complex relationship between the nature of searching (e.g. active/open) and feelings about finding or not finding:

[I’m] *a bit sad. All my unanswered questions are still there. All my wondering about what traits I share with the donor or donor siblings is still theoretical.* (Open to contact, not connected)

Some participants explained that their experiences had changed their expectations:

*…over time, with no connections having yet been made, my expectations have lowered and I have settled into feeling ambivalent about making any connections on the DNA testing sites or through the HFEA. It is a bit disheartening and deflating, to try with no success, and I hope that one day I can make one of these genetic connections I so sorely wish for.* (Open to contact, not connected)

In contrast, for other participants, expectations had increased because of making connections. One participant, who had previously had ‘*zero*’ expectations but had identified a sibling, explained that ‘*my/our expectations were that we had a good chance of finding other donor sibs and* [the] *donor’s other blood relatives either then or as time went on*’ (Active searcher, connected).

Some participants gave mixed responses about their expectations, expressing feelings of hope and optimism along with other feelings:

*As I’ve not yet tried to make connections I don’t feel disappointed. I’m hopeful I will make connections in the future when I’m ready to search… I don’t really have any expectations, other than I don’t think it will be easy or necessarily successful.* (Open to contact, not connected)

*Sad would be my main feeling about this. I feel like I am missing out on time with these connections in my life. I would love to make any genetic connections, donor’s children, donor herself or my half siblings and other genetic relatives. Yet despite feeling sad, disappointed and a little deflated about having no success so far, I still remain optimistic that one day I will find these connections.* (Open to contact, not connected)

Other participants explained their expectations in more neutral terms, stating that ‘*If I don’t find answers, at least I’ve given it a go*’ (Active searcher, not connected); ‘*It’s never something I’ve felt strongly about*’ (Open to contact, not connected); and ‘*I’m happy as I am, any new connections are a bonus*’ (Open to contact, connected).

#### Searching and finding or not finding as catalysing change

Related to the previous subtheme, several participants also explained that searching had acted as a catalyst for change in terms of how they felt about their connections, themselves, and/or their search. One active searcher said that they felt ‘*less positive*’ about their donor siblings once having connected with them, explaining that ‘*I had romanticised it greatly*’. Feelings of relief, disappointment, and disgust about the donor were also mentioned:

[I feel] *reassured that they are a normal person, not a dodgy character!* (Open to contact, connected)

*Now we have found him I would say I’m disappointed and a little disgusted. We never made any kind of contact with him directly because we heard through his brother (who was very friendly and happy to be in contact) that he was just extremely angry and upset that we existed. As far as we know he showed absolutely no empathy at all towards us.* (Active searcher, connected)

Some participants described that searching had made their donor seem ‘more real’, irrespective of the outcome:

*He feels more like a real person now that I’ve started looking. I feel disheartened that nothing significant* [about the donor] *has come from looking.* (Active searcher, connected)

*He is of course more real now rather than just an idea in my mind*. (Active searcher, connected)

Other participants, both those who had received a response and those who had not, emphasized that the process of contacting their donor had been instrumental in changing their feelings:

*I feel a bit better now as I was unsure if I should try to attempt to make contact but once I did it, I felt a bit of relief like the ball is now in her park and I have at least tried to reach out. I’m okay either way (for the moment as I know feelings can change over the years).* (Open to contact, connected)

*I realised the thing most important to me was that he was a ‘good’ man. I didn’t know that I was searching for that until I found him. He was.* (Active searcher, connected)

The experience of searching, whether having resulted in finding connections or not, was also described by some participants as a catalyst for changing feelings, particularly in terms of increasing feelings of curiosity:

*Now I am even more curious about finding other donor siblings, as I have found it to be a positive experience*. (Active searcher, connected)

*I definitely realised contact might be more possible than I thought and that has made me excited and intrigued.* (Open to contact, connected)

*I thought that being open to it would mean I would agree to being ‘found’ and then I would be found, by someone*… *Not being able to find anyone makes me feel more keen to find them.* (Open to contact, not connected)

*It makes me want to find them more. I was neutral before.* (Active searcher, not connected)

In terms of feelings about themselves, some participants reflected that finding connections had ‘*changed my life*’ (Active searcher, connected) and that it had ‘*filled a gap I didn’t know I had*’ (Active searcher, connected).

#### Experiences of other donor conceived people (including donor siblings)

Several participants reflected on the experiences of other donor conceived people in their responses. Making social comparisons was common, with participants who had found and had a positive experience with connections explaining that they felt ‘*lucky*’ (Open to contact, connected) or ‘*fortunate*’ (Active searcher, connected).

Some participants, who had not made connections, explained:

[I feel] *disappointment. Feeling alienated in the donor conceived community. Seems like everyone is swimming in siblings.* (Active searcher, not connected)

*I watched some documentaries and listened to some podcasts about people who searched for a short time and found so many matches, and so I feel disheartened this didn’t happen to me. I have to remind myself that everyone’s search is different, and these successful ones have made it onto TV.* (Open to contact, not connected)

Among those participants who explained they were disappointed to have not yet made connections, some explicitly compared their experience to the future experiences of their donor siblings:

*I hope that in time more people will be able to find me, and my siblings won’t feel the heartache I did as I will be the first one waiting which makes me feel happier.* (Open to contact, not connected)

*I was disappointed to find I was the only one on the register and so if any of my DCS come forward to register knowing they will find me means I have spared them the disappointment I felt.* (Active searcher, not connected)

Participants also reflected on their donor siblings’ thoughts, feelings, and correspondence in different ways:

*I’m angry that either they haven’t been told or that they don’t care about searching for contact*. (Active searcher, not connected)

*I feel* [donor siblings] *want to find the donor and I’m just part of the puzzle to help them do that…* [I] *don’t really feel like there is a connection and I’m ok with that, they found me, I’ve never actively searched.* (Open to contact, connected)

*I have been contacted by other people conceived using the same donor. I am not interested in any kind of contact. It makes me feel bad because I worry they will feel rejected but I am just not interested and they are strangers to me so it isn’t a rejection of them as individuals. I had one who messaged me multiple times after I had said I didn’t want to be contacted. It annoyed me as I had been clear on how I felt, but I do understand that they had very different feelings about being donor conceived and wanting to make connections.* (Not searching, connected)

## Discussion

This study offers an insight into the experiences of donor conceived individuals who are actively searching for their donor connections, those who are open to contact but not actively searching, and those who are not searching or open to contact. Given that most of the literature to date on this topic has tended to focus on donor conceived individuals who are interested in and/or searching for connections (Indekeu *et al.*, 2021), the findings of this study present a more nuanced picture that unpacks both the meaning of searching and its implications among those who are donor conceived. The present findings suggest that not only is there variability in how motivated donor conceived individuals are to search for connections, but also that previously identified factors do not appear to account for this variance. The results of this study, namely that those who are actively searching and those who are open to contact do not differ in terms of their demographic characteristics or in when and how they found out about being donor conceived, therefore complicate the conclusions drawn from previous research (i.e. that factors including the age of disclosure may drive interest in donor connections; Indekeu *et al.*, 2021). The present study’s findings, based on a sample of individuals who were mostly told about their conception in adulthood, show that among this group, there is variability in search behaviour. Findings also offer new insights into the ways that donor conceived people with different degrees of interest in searching for connections may differ, notably in terms of their feelings about genetic information (thus extending the findings of previous research, e.g. Indekeu and Hens, 2019), along with there being differences in the methods they use to search (e.g. genetic testing and social media). That donor conceived people may have different preferences with regards to searching (e.g. whether to search, and the methods with which they do this) was also recently concluded in a qualitative interview study with donor conceived young adults (Zadeh, 2024).

While these findings are indeed noteworthy, they must be read alongside the quantitative and qualitative results of this study about finding, being found by, and not finding donor connections. These experiences are clearly characterized by complexity, irrespective of search status (i.e. active, open, or not searching). Firstly, the topic of searching for and finding donor connections prompts reflection, irrespective of an individual’s search status, and prior to their search activity. Secondly, and relatedly, searching is not a single, one-time event, but rather a process that for many donor conceived people appears to be characterized by uncertainty (indeed, no one single method had resulted in finding connections among all the participants making use of it). In fact, searches may be experienced as open-ended regardless of whether connections have been made (see also Newton, 2023; Zadeh, 2024). Contextually, this is likely because of the long history of donor anonymity in the UK and is also of relevance given the global context of gamete donation, which includes transnational donation and donations outside of the clinical context that are not subject to national regulatory frameworks (i.e. the UK HFEA’s legal limits on the number of families a donor can donate to). Parents’ non-disclosure of donor conception, a practice that, although less common than it was historically, continues today (Lysons *et al.*, 2023), further intensifies the possibility that donor conceived people’s searches for donor connections will remain open-ended.

The findings also suggest that the process of searching may generate feelings or lead to changes in feelings (i.e. increased or decreased curiosity) about connections, irrespective of search status and search outcomes (e.g. whether connections are found or not). Complexity additionally characterizes positive search outcomes, such that where connections are made, there may be uncertainty about the thoughts and feelings of those involved and/or whether and how to proceed in establishing relationships. The implications of the absence of social scripts for what connections mean and how they should be approached, negotiated, and maintained has been addressed in the previous literature (Hertz, 2022; Indekeu *et al.*, 2022). However, it is clear from the present study’s findings that not only are donor connections of varying importance to individuals (see also Newton *et al.*, 2023) but also that these differences in meaning-making about connections are *made visible* when contact (whether desired or not) is made. Findings also show that the outcomes for those who make donor connections differ, both in general, and between different donor connections, aligning with the limited existing literature on donor sibling relationships (Hertz and Nelson, 2020; Indekeu *et al.*, 2022). This means that support for those who have made connections needs to be nuanced (Indekeu *et al.*, 2022) and to account for the potential mismatch in desires and expectations among those who are connected.

In fact, the implications of the present study for practice are significant. The findings suggest that targeting support resources at the point of potential contact between individuals (i.e. through ringfenced funding for intermediary contact services) is unlikely to be most helpful to donor conceived people. Practitioners and policymakers should consider more carefully the possible needs of donor conceived people for earlier intervention, in keeping with the recent guidance of the ESHRE Working Group on Reproductive Donation and Others (2022), for instance, which recommended the provision of counselling at all ages, as also recommended by donor conceived people themselves (Schrijvers *et al.*, 2019; Zadeh *et al.*, 2024). Moreover, support may be required by those who do not desire contact but are found (see also Zadeh, 2024), but these individuals, who do not engage with specific registers or services for making connections through which much support is diverted (Crawshaw *et al.*, 2016; Indekeu *et al.*, 2023), are unlikely to receive it, given these present arrangements. Register-based support is also unlikely to reach those who make connections through different means, such as DNA testing, a method used by the participants in this study more than any other single method for searching for connections. These findings thus also warrant reiteration of the concerns previously raised about the lack of information and support provided by commercial testing websites for donor conceived people (Crawshaw, 2018; Indekeu *et al.*, 2022, 2023; Gilman *et al.*, 2024; Zadeh, 2024). Specific recommendations about the role of peer support in supporting individuals in searching/not searching and finding/not finding donor connections should also bear in mind the present study's findings relating to the social comparisons made by participants between themselves and other donor conceived people.

In terms of the limitations of the study, although the number of participants who did not wish to make connections was small and included three participants who were not looking to make connections because they had already made them, researchers cannot discount the possibility that donor conceived people who are disinterested in donor connections are also unlikely to participate in research on this topic. It is noteworthy that most participants in the study were members of relevant community organizations, and that recruitment mostly proceeded through these groups. Research that adopts a different approach to sampling (e.g. Jadva *et al.*, 2023) would provide a more holistic picture of the perspectives of donor conceived people with regards to searching and not searching for donor connections. Future research could also take a stratified approach to sampling to learn more from donor conceived individuals (e.g. those conceived through oocyte donation, men) about whose experiences little is yet known. However, a strength of the study is that most of its participants were conceived by heterosexual couples, suggesting that the received wisdom based on the limited research that donors and/or others conceived using the same donor are particularly of interest to donor conceived people in single-mother families should be subject to further reflection and empirical scrutiny (see also Casteels *et al.*, 2024). The findings overall should be helpful to policymakers and practitioners in the field who are presently reflecting upon how best to support donor conceived people in the context of searching for donor connections.

## Data Availability

The data underlying this article will be made available via the UK Data Service ReShare repository at the end of the research project in December 2024.
